# Genome-Wide Detection of Copy Number Variations among Diverse Horse Breeds by Array CGH

**DOI:** 10.1371/journal.pone.0086860

**Published:** 2014-01-30

**Authors:** Wei Wang, Shenyuan Wang, Chenglin Hou, Yanping Xing, Junwei Cao, Kaifeng Wu, Chunxia Liu, Dong Zhang, Li Zhang, Yanru Zhang, Huanmin Zhou

**Affiliations:** 1 College of Life Sciences Inner Mongolia Agricultural University, Hohhot, China; 2 Key Laboratory of Biological Manufacturing, Hohhot, China; Auburn University, United States of America

## Abstract

Recent studies have found that copy number variations (CNVs) are widespread in human and animal genomes. CNVs are a significant source of genetic variation, and have been shown to be associated with phenotypic diversity. However, the effect of CNVs on genetic variation in horses is not well understood. In the present study, CNVs in 6 different breeds of mare horses, Mongolia horse, Abaga horse, Hequ horse and Kazakh horse (all plateau breeds) and Debao pony and Thoroughbred, were determined using aCGH. In total, seven hundred CNVs were identified ranging in size from 6.1 Kb to 0.57 Mb across all autosomes, with an average size of 43.08 Kb and a median size of 15.11 Kb. By merging overlapping CNVs, we found a total of three hundred and fifty-three CNV regions (CNVRs). The length of the CNVRs ranged from 6.1 Kb to 1.45 Mb with average and median sizes of 38.49 Kb and 13.1 Kb. Collectively, 13.59 Mb of copy number variation was identified among the horses investigated and accounted for approximately 0.61% of the horse genome sequence. Five hundred and eighteen annotated genes were affected by CNVs, which corresponded to about 2.26% of all horse genes. Through the gene ontology (GO), genetic pathway analysis and comparison of CNV genes among different breeds, we found evidence that CNVs involving 7 genes may be related to the adaptation to severe environment of these plateau horses. This study is the first report of copy number variations in Chinese horses, which indicates that CNVs are ubiquitous in the horse genome and influence many biological processes of the horse. These results will be helpful not only in mapping the horse whole-genome CNVs, but also to further research for the adaption to the high altitude severe environment for plateau horses.

## Introduction

Previous genomic research has suggested that single nucleotide polymorphisms (SNPs) are the main form of genome structure change [1]–[3]. This notion changed in 2004 when two groups of scientists published the first whole-genome maps of copy number variations in seemingly healthy individuals [4], [5]. Copy number variation is a form of structure variation, which has an important influence on phenotype diversity, environmental adaptability and disease susceptibility [6]–[8]. Copy number variant is described as a segment of DNA ranging from 50 bp to several megabases (Mb) that is copy number variable when compared with a reference genome [9], including deletion, insertion, replication and composite multisite mutation. Several studies demonstrate that about 30% of the human genome is affected by CNVs [10]. Further research suggests that, CNV exists not only in the human genome [11]–[13], but also widely in other mammals (pig [14], [15], cattle [16]–[18], sheep [19], [20], mice [21], [22]) and plants [23], [24]. CNVs contain gene coding regions or regulatory elements and may play an important role in gene expression. The effect of CNV on phenotypic diversity on domestic animal has been confirmed [25], [26]. Array comparative genomic hybridization (aCGH) is a validated method to detect the amplified or deleted genome DNA [27], and has already been used for detection of CNVs in many species [12], [15], [19], [20].

Since the 1960’s, with the continuous development of agricultural mechanization, the use of horses as motive power in agriculture has gradually disappeared. Horses however did not fade from human life. In many countries, horses have become domestic animals of social and economic value [28]. Different breeds are the result of the past long-term human and natural selection. Currently, there are more than 200 breeds world-wide. Nevertheless, genetic variation of different phenotypes and biological characteristics among different horse breeds are still relatively unexplored. In 2009, one UK team reported the first high-quality draft sequence of the genome of the horses [29]. Until now, CNV analyses in horses have been performed based on aCGH array [30] and SNP array [31], [32]. But the study by CGH only targeted exons and therefore was not comprehensive and did not identify CNVs in intergenic regions.

Low temperatures, environmental hypoxia and low precipitation are the general severe nature of the plateaus. Through a long-term evolution, Mongolia horse, Abaga horse, Hequ horse, Kazakh horse and other highland horses have adapted to live in these rigorous conditions. They could thrive normally in the highlands. While Debao pony, Thoroughbred and other non plateau horses can not adapt to the plateau’s environment, and in addition have many physiological and phenotypic differences (endurance, body size) from those highland horses. In this research, we investigated genome-wide characteristics of CNVs in 6 horses representing 6 distinct breeds by using the aCGH method and performed GO and KEGG analysis for the CNVs genes. This result is an important complement to the mapping of horse whole-genome CNVs and helpful to study plateau horses’ adaption to the high altitude environment.

## Results and Discussion

### CGH-chip Design and Whole-genome Identification of CNVs

A total of 1,402,459 probes were designed. The minimum spacing of probes was 1101 bp, the maximum spacing was 182,470 bp, and the average distance between probes was 1650 bp. In total, the probes covered 75,991,642 bp across all chromosomes (Table S1 in File S1).

**Figure 1:**
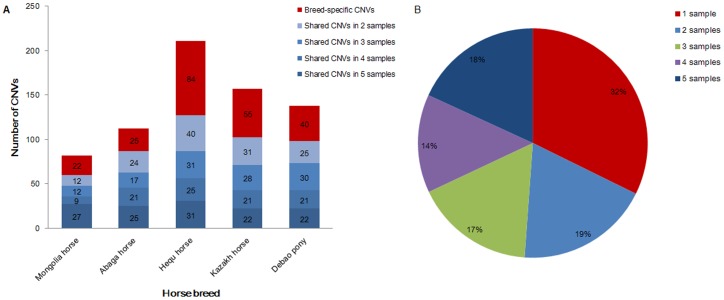
Distribution of CNVs in different breeds. (A) Distribution of breed-specific and shared CNVs among diverse horse breeds. The red represents breed-specific CNVs. The blue represents shared CNVs, and the color from light to dark indicates CNVs shared between 2, 3, 4 and 5 samples. (B) Percentage of CNVs shared among samples. The red represents 1 sample. The blue represents 2 samples. The green represents 3 samples. The purple represents 4 samples. The dark blue represents 5 samples.

Array CGH was performed to identify CNVs among 6 mare horses representing 6 diverse breeds: Mongolia horse, Abaga horse, Hequ horse, Kazakh horse, Debao pony and Thoroughbred. The first four were plateau horses and the last two were not. A single Thoroughbred mare was used as the reference sample. CNVs were analysed on autosomes by comparing the ratio of signal intensities between test samples and the reference. To determine the false positive rate (FPR) of the aCGH, we carried out a self-self hybridization of the reference Thoroughbred. A stringent criterion with the threshold value of 0.5 was used to reduce the FPR of CNV calling, and there was no CNV identified in the self-self hybridization. Therefore, we demonstrated that the result of the aCGH chip technique employed in this study to identify CNVs was reliable. In total, 700 CNVs on autosomals were detected (Table 1 and Table S2 in File S1), including 302 gains and 398 losses. And the average number of CNVs per individual was 120, ranging from 82 to 211, slightly less than Doan of 139.3 (2368/17, CGH analysis) [30], while significantly greater than Dupuis of 5.9 (2797/477, SNP analysis) [31] and Metzger of 5.6 (4013/717, SNP analysis) [32]. This might be caused by different experimental samples or detection methods of CNV. The lengths of CNVs ranged from 6.1 Kb to 0.57 Mb with an average of 43.08 Kb and a median of 15.11 Kb. The mean size of CNVs was low compared to the previous researches in horses (99.4 Kb, 229 Kb and 368.72 Kb). CNVs were not distributed across all chromosomes, none appearing on chromosomes 30 and 31. We defined any CNVs detected only in a single horse and not shared with another animal in this study as the breed-specific CNVs, with the number of per breed ranging from 22 to 84. Any CNVs that identical or overlapped between two or more horses were defined as shared CNVs, the number varying from 60 to 127 (Table 1). We found that each horse shared most of their CNVs with at least another animal, and approximately 68% of the CNVs were detected in at least 2 individuals (Figure 1A, B). We didn’t detect the gains and losses in the reference Thoroughbred horse, the CNVs present in this sample would affect the results of the other breeds studied and may caused an increase in the number of shared CNVs. A total of 518 genes within or overlapped with the CNVs were identified (Table S3 in File S1), comprising about 2.26% of all the horse genes. And the number of genes within or overlapped with CNVs in each species varied from 62 to 161.

**Table 1:** The number of detected CNVs with the reference of Thoroughbred.

Breed	Types	Sex	CNVs	Gains	Losses	Average size of CNVs (Kb)	Genes
Mongolia horse^1^	Plateau	F	82 (22)	42 (4)	40 (18)	33.66^a^ (12.70^b^)	62 (7)
Abaga horse^2^	Plateau	F	112 (25)	54 (8)	58 (17)	40^a^ (15.67^b^)	98 (20)
Hequ horse^3^	Plateau	F	211 (84)	91 (21)	120 (63)	39.11^a^ (22.62^b^)	161 (61)
Kazakh horse^4^	Plateau	F	157 (55)	54 (13)	103 (42)	52.08^a^ (33.01^b^)	134 (31)
Debao pony^5^	Non Plateau	F	138 (40)	61 (13)	77 (27)	47^a^ (16.84^b^)	124 (28)
Total	–	–	700 (226)	302 (59)	398 (167)	43.08 (22.39)	518

Numbers in parentheses indicate breed-specific CNV.

a, b *P*>0.05, no significant difference in the average size of CNVs per individual.

*P*<0.05, significant difference in CNV status between 1 and 4, 2 and 4.

*P*<0.05, significant difference between gain and loss in 3 and 4.

### The Detailed Features of CNVRs in the Horse Genome

**Figure 2:**
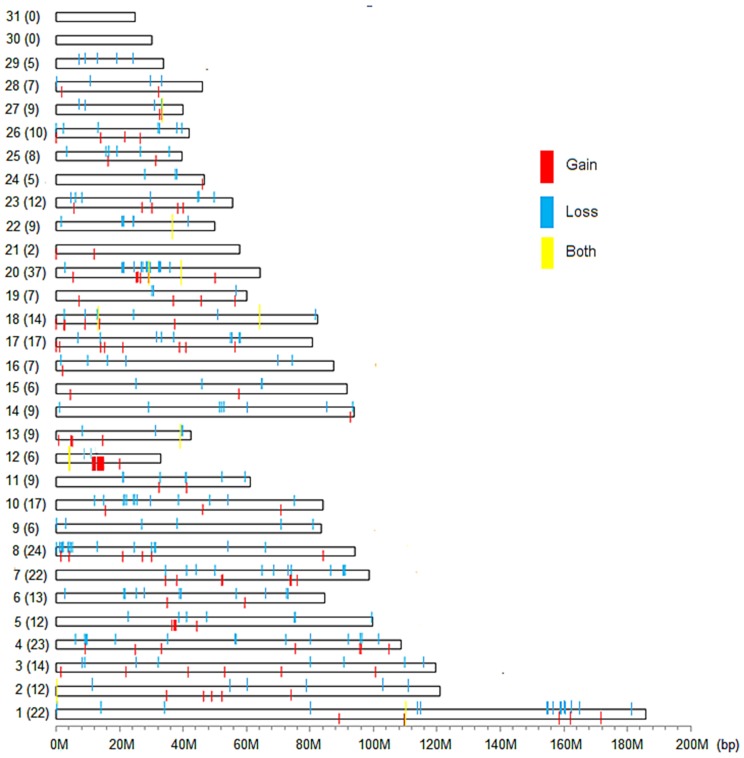
Distribution of CNVRs in the horse genome. Numbers in parentheses represent CNVRs. The red represents gain CNVRs. The light blue represents loss CNVRs. The yellow represents both CNVRs.

After merging the overlapping CNVs, a total of 353 CNVRs were identified (Figure 2 and Table S4 in File S1) with 109 (30.88%) CNVRs called as gains, 234 (66.29%) called as losses and the remaining 10 (2.83%) called as both (gains and losses). Comparisons between our detection results of CNVRs with recent CNVs studies showed that the number of CNVRs identical or overlapping were 93 (26.35%), 59 (16.71%), and 15 (4.25%) with Doan [30], Dupuis [31] and Metzger [32], respectively (Table S5 in File S1). We found that our identified CNVRs had the highest concordance with Doan, which could be due to the same test method of CGH. In the comparison with the results of Metzger, only 15 overlapped CNVRs were found, we proposed that the difference could be due to the different research platforms, or the application of more stringent quality criteria and combination of three detection algorithms in Metzger’s research. In total, 9 CNVRs were detected by all CNVs studies. The length of the 353 CNVRs ranged from 6.1 Kb to 1.45 Mb with average and median sizes of CNVRs of 38.49 Kb and 13.1 Kb, respectively. The average size of CNVRs on each chromosome ranged from 7.61 Kb to 435.23 Kb (Table 2). These CNVRs spaned 13.59 Mb of the horse genome and accounted for approximately 0.61% of the genome sequence. This result was shorter than Li Jiang and Joao Fadista’s detection in bovine [16], [18], and also shorter than Doan’s finding in horses [30] and Yan Li’s identification in pigs [15]. We speculated that could be due to the different genetic background of the selection of animals or research platforms. And then we also assessed these CNVRs commonly affected in all experimental breeds, a total of 41 CNVRs were identified, with ranging from 4.8 Kb to 268 Kb (Table S6 in File S1).

**Table 2:** Chromosome distribution of CNVRs in horses.

Chr	No. of CNVRs	No. of genes	Length of CNVRs (bp)	Length ofchromosomes (bp)	Percentage (%)	Average of CNVRs(Kb)
1	22	59	1486385	185838109	0.8	67.56
2	12	11	471323	120857687	0.39	39.28
3	14	8	339820	119479920	0.28	24.27
4	23	11	685515	108569075	0.63	29.81
5	12	12	407652	99680356	0.41	33.91
6	13	8	188057	84719076	0.22	14.47
7	22	48	940910	98542428	0.95	42.77
8	24	30	1034813	94057673	1.1	43.12
9	6	3	142125	83561422	0.17	23.69
10	17	18	338576	83980604	0.4	19.92
11	9	12	161560	61308211	0.26	17.95
12	6	171	2611353	33091231	7.89	435.23
13	9	13	196374	42578167	0.46	21.82
14	9	4	291428	93904894	0.31	32.38
15	6	1	77346	91571448	0.08	12.89
16	7	3	131240	87365405	0.15	18.75
17	17	2	194530	80757907	0.24	11.44
18	14	8	410207	82527541	0.5	29.3
19	7	6	138162	59975221	0.23	19.74
20	37	48	1526667	64166202	2.38	41.26
21	2	3	78028	57723302	0.14	39.01
22	9	4	265150	49946797	0.53	29.46
23	12	3	173586	55726280	0.31	14.47
24	5	1	89110	46749900	0.19	17.82
25	8	10	271071	39536964	0.69	33.88
26	10	8	251810	41866177	0.6	25.18
27	9	6	176564	39960074	0.44	19.62
28	7	4	469128	46177339	1.02	67.02
29	5	3	38069	33672925	0.11	7.61
30	0	0	0	30062385	0	0
31	0	0	0	24984650	0	0
X	–	–	–	124114077	–	–
Total	353	518	13586559	2,367,053,447	0.61	38.49

**Figure 3:**
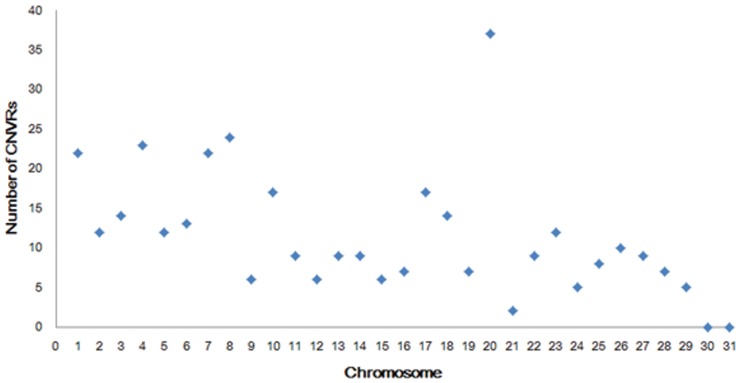
Chromosome distribution of the number of CNVRs.

**Figure 4:**
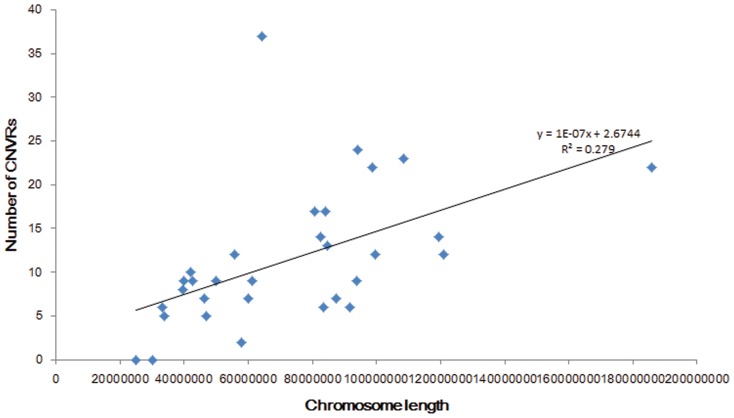
Correlation between the number of CNVRs and chromosome length.

**Figure 5:**
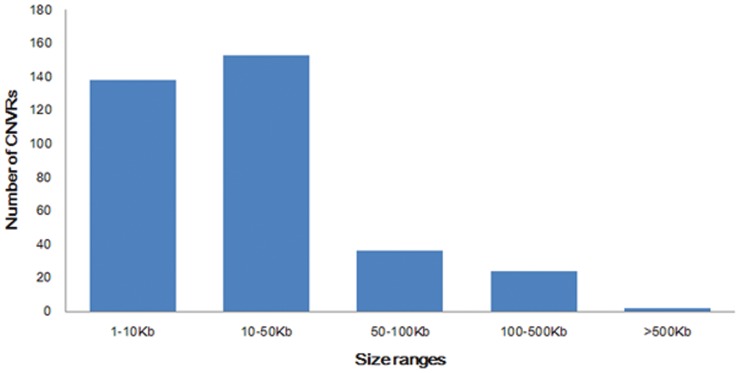
Size range distribution of the CNVRs detected.

Through the comparison of the average length of gain CNVRs (45.92 Kb) and loss CNVRs (33.87 Kb), we found gain regions had slightly larger sizes than loss regions (ANOVA not statistically significant at *P*>0.05, Table 3). This was similar to the previous research results that losses were under stronger purifying selection than gains [33]. The differences in CNVRs numbers per chromosome were very significant (Figure 3), ranging from 2 of chromosome 21 to 37 of chromosome 20. There was no correlation between the incidence rate of CNVRs and the length of chromosomes (Figure 4), such as the longest chromosome 1 only cotained 22 CNVRs detected but chromosome 20 (1/3 length of chromosome 1) included the largest number of 37 CNVRs. The scales of CNVRs on each chromosome were extremely different. Chromosomes 8, 12, 20, 28 had the dense CNVRs covering more than 1% of genomic sequences, and the CNVRs of chromosome 12 covered 7.89% sequences of the chromosome,especially, wheras the coverage percent on chromosome 15 was only 0.08%. This demonstrated that the distribution of CNVRs on chromosomes was not uniform. We also detected 6 CNVRs with 171 genes on chromosome 12, while 37 CNVRs only containing 48 genes on chromosome 20, which appeared that chromosome 12 aberrations were in more gene dense regions. The length of CNVRs were divided into five regions of 1–10 Kb, 10–50 Kb, 50–100 Kb, 100–500 Kb and >500 Kb (Figure 5). Generally, the more probes used ( = bigger length) the easier was to find a CNV. So the bigger the CNV was, the easier it was to detect it. While it should be noted that 291 (82.44%) CNVRs were less than 50 Kb, and only 2 (0.57%) CNVR >500 Kb in size were detected. This finding could be due to the fact that shorter CNVRs were more prevalent in horse genome, what was similar with other studies [17], [19].

**Table 3:** Characteristics of the CNVRs.

Status	No. of CNVRs	Length of CNVRs (bp)	Average of CNVRs (Kb)	Total length of CNVRs (bp)	Percentage (%)
Gains	109	5005156	45.92^c^	13586559	36.84
Losses	234	7924489	33.87^c^		58.33
Both	10	656914	65.69^c^		4.84

c *P*>0.05,no Significant difference among the average of different status CNVRs.

### Gene Contents of Horse CNVs

**Figure 6:**
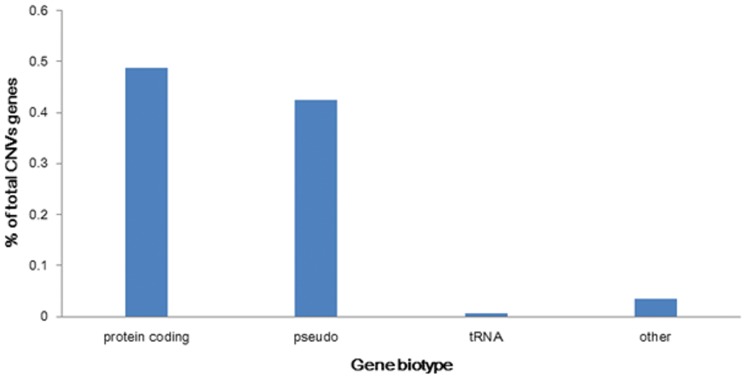
Percentage of gene biotypes affected by CNVs.

In order to analyze the genes in CNVs and understand the potential effects of CNVs on various biological processes, we performed functional analysis clustering of these genes affected by CNVs to understand the potential effects of CNVs on gene biotypes in horses. Two hundred and sixty five (51.16%) protein coding genes, 231 (44.59%) pseudo genes, 3 (0.58%) tRNA genes and 19 (3.67%) other genes were found (Figure 6). One hundred and sixty six (47.03%) CNVRs encompassed one or more genes, however 187 (52.97%) CNVRs didn’t involve any genes. In order to determine the likely biological effects of these genes, we performed Gene Ontology (GO) analysis for these CNVs genes. Because to the horse genome was poorly annotated in the GO database, we converted all Gene Symbol IDs to the human ortholog Gene Symbol IDs (Table S3 in File S1). Functional annotation analysis was performed with the DAVID bioinformatics resources v6.7 (Accessed Mar 2013, (http://david.abcc.ncifcrf.gov/home.jsp). GO analysis revealed that CNVs genes belonged to these classes of genes that participated in olfactory receptor activity,sensory perception, cognition, G-protein coupled receptor protein signaling pathway, neurological system process, cell surface receptor linked signal transduction, plasma membrane, integral to membrane, intrinsic to membrane and other basic metabolic processes (*P*<0.05 - Table S7 in File S1). The Kyoto Encyclopedia of Genes and Genomes (KEGG) pathway analysis revealed that these genes were mainly represented in the pathway of olfactory transduction (*P*<0.05 - Table S8 in File S1). This result was similar with these findings in human, pigs and cattle.

Through the GO analysis for these CNVs genes, we found 7 genes (CYP4A11, CYP4X1, EIF2AK1, CYP2C18, CYP4F22, NOS2, CYP4B1) were related to the heme binding (*P*<0.014), which correspond to the horse Gene Symbol IDs: LOC100630182, LOC100630164, LOC100062309, LOC100061400, LOC100063353, LOC100146437, LOC100629469, LOC100064176, and the majority were only in the plateau horses breed-specific CNVs (except LOC100629469) of which most existed in Hequ horse (Table 4). Hequ horse was one breed of the Chinese highland horses distributing in the eastern of Tibetan Plateau, where the altitude was above 4000 m. They could adapt well to the harsh nature of the plateaus of low temperatures, environmental hypoxia and low precipitation through long-term evolution. And then we interrogated our data with annotations from the KEGG Pathway Database (Accessed Dec 2013, (http://www.genome.jp/kegg/pathway.html), we found 4 genes (CYP2C18, CYP4A11, NOS2, EIF2AK1) involved in “Retinol metabolism”, “HIF-1 signaling pathway”, “Protein processing in endoplasmic reticulum”, etc. The changes in oxygen utilization may be the key for the adaption to high altitude. From the gene ontology and genetic pathway analyses, we hypothesize that these genes in CNVs may have some relation with the adaption to the severe environment associated with plateaus in China for plateau horses. Further studies are required to assess the function of these genes in horses.

**Table 4:** The information of 7 genes.

Gene Symble	Homologous Human Gene Symbl	Region	Status	Involve horse breeds
LOC100061400	CYP2C18	chr1∶33918586,33937152	loss	Kazakh horse
LOC100064176	CYP4B1	chr2∶11618958,11710183	loss	Hequ horse
LOC100630164	CYP4X1	chr2∶11618958,11710183	loss	Hequ horse
LOC100630182	CYP4A11	chr2∶11618958,11710183	loss	Hequ horse
LOC100146437	NOS2	chr11∶41831456,41849869	gain	Hequ horse
LOC100062309	EIF2AK1	chr13∶1497390,1508926	gain	Abaga horse
LOC100063353	CYP4F22	chr21∶744418,785384	gain	Hequ horse, Abaga horse

### DGV and OMIM Analysis

We also queried for the horse-human ortholog genes with the Human Database of Genomic Variants (DGV, Accessed Apr 2013, (http://projects.tcag.ca/variation/) and 275 genes were found (Table S3 in File S1). In the end, to analyze whether the CNVs affecting genes were associated with disease, the horse-human ortholog genes were imported in Online Mendelian Inheritance in Man (OMIM, Accessed Apr 2013, (http://omim.org/), of which 96 had been associated in human disease (FSHD, Coffin-Siris syndrome, Bardet-Biedl syndrome 3, Cataract, autosomal recessive congenital 4, Blood group, ABO system, etc - Table S9 in File S1).

### Validation of CNVs by qPCR

Quantitative PCR (qPCR) was performed to validate 5 CNVRs chosen from the CNVRs detected by aCGH. These CNVRs represented different status of copy number variations (gain and loss) of which all contained functional genes. Two of these CNVRs contained CYP4A11 (LOC100630182) and EIF2AK1 (LOC100062309), respectively. Among every breed, 5 or 6 different individuals were performed qPCR test. And results showed that, 89.69% of the qPCR results were consistent with the aCGH chip (Table S10 in File S1). Thus, qPCR results proved that the detection results of aCGH chip were credible, and also demonstrated discrepancy existed among the CNVs of different individuals of the same species.

## Conclusions

In summary, we described a map of Chinese horse CNVs by a high-resolution aCGH, which was confirmed to be a valid method to detect animal genome-wide CNVs. In total, we indentified 700 CNVs, grouped into 353 CNVRs, which accounted for approximately 0.61% of the horse genome sequence. GO analysis and comparation of CNV genes among different breeds demonstrated that some genes related to heme binding could have effect on the adaption to the plateau severe environment for horses. Through the validation of CNVRs by qPCR, the detection results of aCGH chip were low error rate. This study was the first report of copy number variations in Chinese horses, which will be helpful not only in mapping the horse whole-genome CNVs but also further study to the adaption to the plateau’s harsh nature for plateau horses.

## Methods

### Sample Preparation

The whole study protocols for collection of the tissue samples of experimental individuals were reviewed and approved by the Agricultural Hall of China Inner Mongolia.

The study population consisted of Mongolia horse (Inner Mongolia, n = 1, ♀), Abaga horse (Inner Mongolia, n = 1, ♀), Hequ horse (Gansu Hequ, n = 1, ♀), Kazakh horse (Xinjiang Province, n = 1, ♀), Debao pony (Guangxi Debao, n = 1, ♀) and Thoroughbred (Beijing, n = 1, ♀). In this study, a total number of 6 individuals were chosen for CGH and they were divided into two types according to the altitude of their distributions (Table 1). We used the jugular vein blood sampling after the horse was injected with tranquillizers, blood, 15 mL, was collected in 50 mL centrifuge tube with EDTA anticoagulant, and then preservated at −80°C. Genomic DNA was extracted from blood using AxyPrep Blood Genomic DNA Maxiprep Kit and purified by Wizard® Genomic DNA Purification Kit according to the manufacturer’s instructions. The 200 ng genomic DNA was analyzed on 1% agarose gel to ensure there were no signs of RNA contamination or degradation.

### Array CGH Processing and Analysis

This study used a customized *Equus caballus* CGH 3×1.4 M Whole-Genome Tiling (NimbleGen), which was designed based on the Thoroughbred genome sequence published on NCBI Database in October 2007 by The Genome Assembly Team (Accessed Nov 2012, (http://www.ncbi.nlm.nih.gov/genome/145). The average distance between probes was 1650 bp, and through the SSAHA algorithm to select specific probes.

Labeling, hybridization, washing, array scanning and data analysis were carried out according to the NimbleGen CGH Arrays User’s Guide and performed at CapitalBio Corporation (Beijing, China).

Briefly, pairs of genomic DNA (500 ng) were labeled with fluorescent dyes Cy3 (test samples) or Cy5 (reference DNA), samples were co-hybridized to *Equus caballus* CGH 3×1.4 M Whole-Genome Tiling, with a median probe spacing of 1650 bp.

The arrays were scanned using MS200 scanner (NimbleGen) with 2 µm resolution, and fluorescent intensity data was extracted with NimbleScan 2.6 software. The hybridization control (STC, Sample Tracking Controls) were used to confirm that the correct sample was hybridized to each array.

For each spot on the array, log2 ratios of the Cy3-labeled test sample versus Cy-5 reference sample were computed. Before normalization and segmentation analysis, spatial correction was applied, which corrected position-dependent non-uniformity of signals across the array, specifically, locally weighted polynomial regression (LOESS) is used to adjust signal intensities based on X, Y feature position [34]. Normalization was then performed using the q-spline method [35], compensated for inherent differences in signal between the two dyes. This was followed by segmentation using the CNV calling algorithm segMNT [36]. The segMNT algorithm identified copy number variation using a dynamic programming process that minimizes the squared error relative to the segment means, which showed better performance than the DNACopy algorithm [37]. The segments with |mean log_2_ ratio| ≥0.5 and at least 5 consecutive probes were retained [38]. Log_2_ratio means of all probes on a segment were used to classify the segment as “gain”, “unchanged” and “loss” with following criteria: |log_2_ ratio|<0.5 represented “unchanged”; log_2_ ratio≥0.5 represented “gain”; log_2_ ratio≤-0.5 representsted “loss”. And further visualization with SignalMap software (NimbleGen).

### Enrichment Analysis

Gene contents of the identified CNVRs were retrieved from the NCBI Genome Database based on the EquCab2.0 sequence assembly. Due to the horse genome being poorly annotated in the GO database, we converted all Gene Symbol IDs to the human orthologs Gene Symbol IDs. To determine their functional enrichment, we performed GO classification [39] and KEGG pathway annotation [40] of these CNVs genes with the DAVID tool [41]. We also compared these horse-human ortholog genes with the CNV related genes reported in the DGV [42]. By the end, querying for these horse-human ortholog genes with OMIM to detect CNVs affecting genes associated with disease.

### Quantitative PCR

We used the qPCR method to validate CNVRs in this study. The instrumentation was Roche LightCycler® 480,analysis software was Roche LightCycler® 480 Detection System. The primers were designed using the Primer Premier 5 software. A total of 5 pairs of primers covering 5 candidate CNVRs were synthesisesd plus one pair of control primers in the GAPDH gene [30] (Table S11 in File S1). All PCR primers were designed based on its reference sequence in NCBI. All qPCR reactions were performed by using SYBR Green method. The reaction system was 20 µL, which contained 20 ng gDNA, 0.4 µL (10 µM) of both forward primer and reverse primer, 10 µL SYBR Premix Ex Taq II and water. The cycling conditions consisted of 1 cycle at 95°C for 5 min, followed by 40 cycles at 95°C for 60 sec, 60°C for 40 sec, and 72°C for 60 sec, with fluorescence acquisition at 72°C in single mode. All PCRs were performed in 96-well clear reaction plates (Roche Applied Science). We used the relative quantitative method to test samples relative copy number analysis. The value of crossing thresholds (Ct) was composed of three repeated test averaging, and with normalization of control primer (GAPDH). Finally, the relative copy number of the test samples gDNA CNVR were calculated by 2^−ΔΔCt^ [43].

### Data Availability

The full data set and designs from the oligo array CGH experiments have been submitted to GEO [44] under the accession ID: GSE52504.

## Supporting Information

File S1 **File includes Tables S1–S11.** Table S1: The information of aCGH chip designed by Roche NimbleGen. Table S2: The detailed features of CNVs on chromosomes identified in this study. Table S3: Gene contents of CNVs. Table S4: The detailed features of CNVRs on chromosomes identified in this study. Table S5: CNVRs identified in this study in comparison with the previous studies. Table S6: CNVRs commonly identified in all experimental breeds. Table S7: Gene Ontology (GO) analysis of CNVs genes. Table S8: KEGG Pathway analysis of CNVs genes. Table S9: The details of horse-human orthologs genes exist in OMIM. Table S10: The information of quantitative PCR. Table S11: The information of primers used in quantitative PCR.
(XLSX)
